# Duloxetine and pregabalin: High-dose monotherapy or their combination?—Study protocol for a randomized trial in patients with postoperative pain after total knee arthroplasty based on the classification of central sensitization inventory scores

**DOI:** 10.1371/journal.pone.0334400

**Published:** 2025-10-17

**Authors:** Haodong Wu, Shuxin Yao, Yuanchi Huang, Chao Xu, Jianbing Ma

**Affiliations:** 1 Department of Knee Joint Surgery, Honghui Hospital, Xi’an Jiaotong University, Xi’an, China; 2 Medical College of Yan’an University, Yan’an University, Yan’an, Shannxi, China; 3 Department of Health Statistics, Faculty of Preventive Medicine, the Air Force Military Medical University, Xi’an, China; University of Naples Federico II: Universita degli Studi di Napoli Federico II, ITALY

## Abstract

**Background:**

Residual pain after total knee arthroplasty (TKA) often causes patient dissatisfaction. Patients with preoperative central sensitization (CS) are especially susceptible to chronic pain after TKA. Although duloxetine and pregabalin are known to relieve pain in CS patients, there is limited evidence on the precise effectiveness and safety of increasing the dosage or combining these medications. To address this gap, we designed a randomized, triple-blind clinical trial to assess the efficacy and safety of increasing the maximum dosage or combining these drugs for patients who do not respond to standard doses.

**Methods:**

Patients scheduled to undergo primary unilateral TKA will be screened for CS using the central sensitization inventory (CSI). CS patients will then be randomly assigned to Groups 1–5, while non-CS patients will be assigned to Group 6. All groups will receive multimodal analgesia. Groups 1 and 6 will receive a placebo. During the initial 6-week period, Groups 2 and 3 will take 60 mg/day of duloxetine, while Groups 4 and 5 will take 300 mg/day of pregabalin. Subsequently, non-responders will enter a 6-week period of high-dose/combination therapy. Group 2 will receive 120 mg/day of duloxetine, Group 3 and 4 will receive a combination of 60 mg/day of duloxetine and 300 mg/day of pregabalin, and Group 5 will receive 600 mg/day of pregabalin. The primary outcome will be to compare residual pain intensity at 6 months between the high-dose monotherapy groups (Groups 2, 5 pooled) and the combination therapy groups (Groups 3, 4 pooled), which will be measured using the brief pain inventory (BPI) 24-hour average pain change. Secondary outcomes will assess pain and functionality.

**Discussion:**

This study aims to evaluate the efficacy and safety of increasing medication to the highest dose or combining two medications in patients with CS who have not responded well to standard doses of duloxetine or pregabalin after TKA. The goal is to provide clinicians with evidence-based recommendations for choosing an appropriate pain management strategy for these patients.

**Trial registration:**

Chinese Clinical Trial Registry, ChiCTR2400081990. Registered on 18 March 2024.

## 1. Introduction

Total knee arthroplasty (TKA) remains a reliable and well-established surgical intervention for treating advanced stages of knee osteoarthritis (OA) [1–3]. Over recent decades, notable progress has been made in the field of TKA, marked by refinements and innovations across surgical techniques, implant design, patient selection criteria, and comprehensive perioperative care. Consequently, the efficacy of TKA has improved, broadening the scope of patients who can benefit from this therapy [4–9]. Nevertheless, around 20% of TKA recipients remain dissatisfied with their clinical outcomes, primarily due to inadequate postoperative pain management [10–12].

Recent studies have revealed that sustained and intense injurious stimuli can trigger peripheral injurious receptors, inducing plastic modifications within the central nervous system (CNS). This phenomenon, termed central sensitization (CS) [13], involves an amplification of neural signaling within the CNS, resulting in pain hypersensitivity or persistent pain even after the removal of injurious stimuli [13–15]. CS is not universally present in all patients with knee OA; however, approximately 20% to 40% of those with advanced OA of the knee develop CS due to chronic pain [16–19]. Moreover, research indicates a significant association between CS and poorer clinical outcomes following TKA [20]. Patients with preoperative CS are more prone to experiencing persistent pain after surgery compared to those without CS [21–24]. Therefore, preoperative screening for CS and implementing appropriate measures may prove effective in reducing residual pain levels and enhancing patient satisfaction post-TKA.

Duloxetine (Cymbalta) is a potent reuptake inhibitor of serotonin and norepinephrine [25]. Serotonin and norepinephrine may regulate the CNS, impacting the descending inhibitory pain pathways. The imbalance of these neurotransmitters is closely linked to chronic pain associated with CS [26–28]. Duloxetine has shown comparable analgesic effects for pain relief in both depressed and non-depressed patients [29]. It has been validated for relieving CNS-mediated musculoskeletal pain, especially chronic knee OA pain [30]. Studies have demonstrated that using duloxetine during the perioperative period effectively reduces morphine consumption in the 48 hours following TKA, with no significant adverse events observed during clinical trials [31]. Moreover, in patients with CS who have undergone TKA, duloxetine reduces pain, decreases opioid use, and enhances recovery without increasing the risk of adverse events [32].

Pregabalin, a medication that modulates the α2-δ-calcium channel subunits, works by reducing Ca2 + influx during depolarization and decreasing the release of excitatory substances from the dorsal horn of the spinal cord [33]. It is commonly prescribed and included in multimodal analgesia protocols [33,34]. Pregabalin belongs to the class of gamma-aminobutyric acid (GABA) agonists, with GABA being a major inhibitory neurotransmitter in the CNS. Decreased GABA neurotransmission is crucial in the etiology and maintenance of CS [35]. Perioperative administration of pregabalin has been shown to reduce the occurrence of chronic neuropathic pain following TKA, decrease opioid consumption, and improve the range of motion of joints [36].

Previous studies have demonstrated that the use of standard doses of duloxetine (60 mg/day) and pregabalin (300 mg/day) alone can significantly alleviate pain in approximately 40% of chronic pain patients [29,37–40]. For those who do not respond fully to monotherapy, compared to the maximum dose of monotherapy, the combination of standard doses of duloxetine and pregabalin may exhibit more significant therapeutic effects in relieving pain and improving drug tolerance [41–43]. Given that duloxetine and pregabalin possess distinct pharmacological mechanisms of action and may complement each other [25,33], the concurrent use of these two drugs in patients with CS undergoing TKA may yield a synergistic effect. Such a synergistic effect has the potential to produce more notable impacts on the alleviation of postoperative chronic pain, acceleration of recovery processes, and enhancement of patient satisfaction. However, currently, there is a lack of sufficient clinical evidence to conclusively demonstrate the effectiveness and safety of this medication strategy in TKA patients.

This protocol aims to address a common clinical question: “In patients with inadequately relieved pain following TKA associated with CS, is it more beneficial to increase the dosage of monotherapy or to initiate early combination therapy with another drug?” For patients who do not respond well to treatment with standard doses of duloxetine or pregabalin for postoperative pain, this protocol will compare the efficacy and tolerability of the maximum dose of monotherapy (duloxetine 120 mg/day or pregabalin 600 mg/day) with a fixed combination of the standard doses of duloxetine (60 mg/day) and pregabalin (300 mg/day) [44]. Additionally, this protocol will directly compare the effectiveness of duloxetine (60 mg/day) versus pregabalin (300 mg/day) during the initial six-week pain management phase. Meanwhile, it will also investigate the differential effects of routine multimodal analgesia regimens on pain control between patients with and without CS. We hypothesize that the combined use of duloxetine and pregabalin will be more effective than high-dose monotherapy in patients with CS who have poor pain control following TKA without compromising postoperative functional recovery or increasing the risk of adverse events.

## 2. Methods

### 2.1. Study design

**Fig 1:**
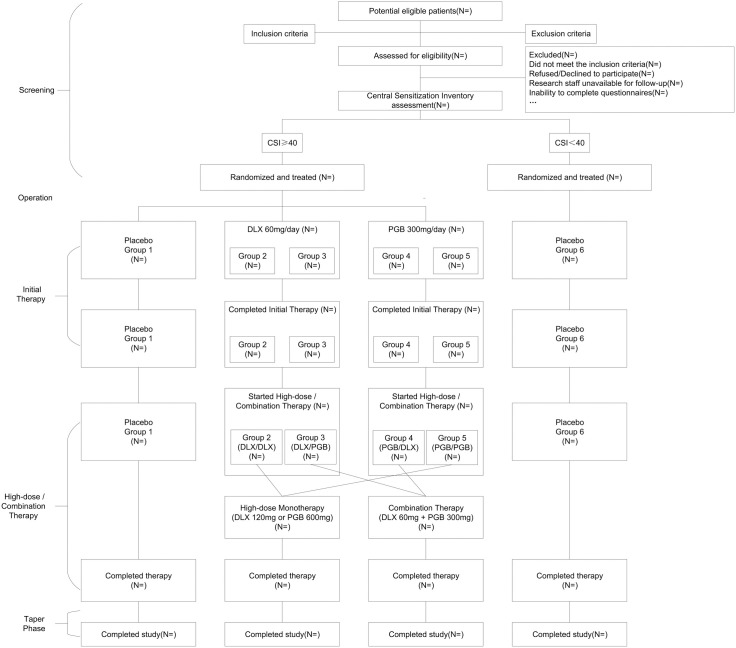
Study assessment schedule (Standard Protocol Items: Recommendations for Interventional Trials (SPIRIT) Figure). BPI: Brief pain inventory; SF-36: 36-Item Short Form Health Survey; WOMAC: Western Ontario and McMaster Universities Osteoarthritis Index; ICOAP: Intermittent and Constant Osteoarthritis Pain Questionnaire; ROM: Range of motion; CSI: Central sensitization inventory; HMAD: Hamilton Depression Rating Scale; PD-Q: PainDETECT questionnaire; CGI-I: Clinical global impression of improvement; PGI-I: Patient Global Impression of Improvement.

This investigation is designed as a prospective, randomized controlled trial with a triple-blind protocol. The study will be conducted at an academic hospital in China: Honghui Hospital, Xi’an Jiaotong University. This protocol adheres to the standard protocol items: recommendations for interventional trials (SPIRIT) 2013 guidelines, including the checklist and diagram (Fig 1), and describes the study design, methods, and statistical analysis plan; results will be published separately upon trial completion [45].

This protocol was approved by the Independent Ethics Committee of Honghui Hospital, the Affiliated Hospital of Xi’an Jiaotong University (No. 202403056) and registered (registration date: 18 March 2024) at Chinese Clinical Trial Registry (http://www.chictr.org.cn, identifier: ChiCTR2400081990). Details of the study will be explained thoroughly to the potential participants by the study investigator. The informed consent form must be signed and provided by all eligible individuals before enrollment. Participants may withdraw from the study at any time without any reason. The participants can withdraw from the study without any reason at any time. This trial does not involve collecting biological specimens for storage. The privacy of all participants will be strictly protected. Any confidential information, such as name, identification number, hospital administration number, etc. will not be exported, and data anonymity will be executed during the process of data analysis and management.

**Fig 2:**
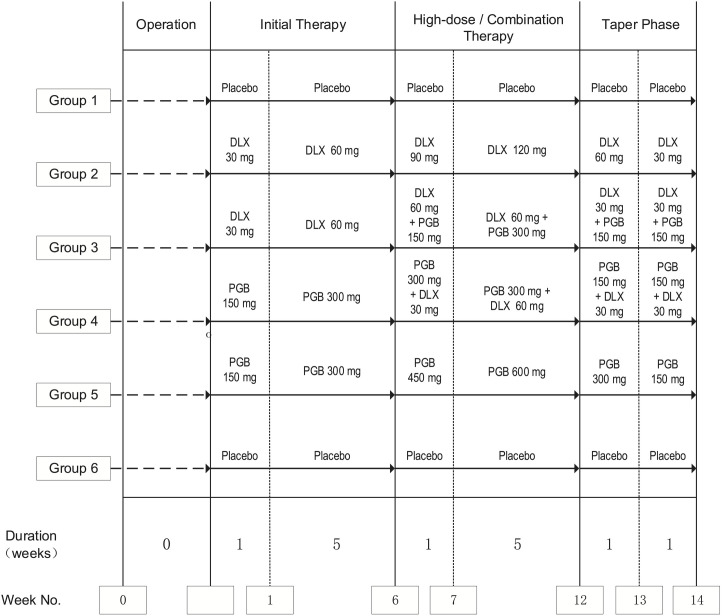
Patient disposition flow chart. CSI: Central sensitization inventory; DLX: duloxetine; PGB: pregabalin.

Eligible candidates for primary unilateral TKA will be enrolled and assessed based on a rigorous set of predefined inclusion and exclusion criteria. The study is planned to commence in August 2024, with a projected recruitment phase exceeding 12 months, to be succeeded by a comprehensive 12-month follow-up period for the enrolled participants. Adhering to the principles outlined in the SPIRIT 2013 guidelines, the protocol for this study has been meticulously formulated to guarantee both clarity and scientific rigor in the design and execution of the trial [45]. The trial flow chart is shown in Fig 2.

### 2.2. Study population

Within the scope of this study, a skilled investigator who is proficient in clinical research practices and possesses the necessary medical qualifications will be responsible for identifying potential candidates from a group of consecutively recruited individuals who have agreed to participate. The selection of participants for the trial will be carefully carried out by applying established inclusion and exclusion criteria to ensure their suitability for enrollment. The process of obtaining informed consent will be conducted by this trained researcher, who is knowledgeable about good clinical practice (GCP) standards. This will protect the ethical integrity of the study and ensure compliance with all regulatory requirements.

### 2.3. Inclusion criteria

To ensure the rigor and appropriateness of participant selection in this investigation, the following inclusion criteria have been meticulously established:

1. Eligible participants must have a diagnosis of knee OA, confirmed through clinical evaluation and imaging studies, and must be scheduled for their primary TKA;
2. Participants must be aged fifty years or older;
3. They should possess clear communication capabilities to facilitate precise conveyance and comprehension of study-related information;
4. The physical status of the participants should be classified between Class I and Class III, according to the American society of anesthesiologists (ASA) physical status classification system;
5. Voluntary participation is mandatory, with participants providing signed informed consent.

### 2.4. Exclusion criteria

To maintain the integrity and safety of the study, participation will be precluded for individuals meeting any of the following exclusion criteria:

1. History of other surgical interventions (e.g., hip, major thoracic or abdominal surgery) within the past year;
2. Prior open surgery on the same side of the knee joint;
3. Intra-articular injections or arthroscopy of the surgical knee within the last three months;
4. Planned contralateral TKA or any other surgical procedures during the study period;
5. Severe preoperative comorbidities anticipated to result in hospitalization or significant impairment of study participation;
6. Documented history of significant peripheral nerve injury;
7. Have neurological disorders or cognitive impairments affecting questionnaire completion (e.g., Alzheimer’s disease, dementia);
8. Allergy to sulfa drugs;
9. History of alcohol or substance abuse or dependence within the last five years preceding enrollment;
10. History of arrhythmias, heart failure, myocardial infarction, or cardiac dysrhythmias at the time of enrollment;
11. Uncontrolled hypertension with systolic blood pressure >180 mm Hg or diastolic blood pressure >110 mm Hg at screening;
12. History of glaucoma (or elevated intraocular pressure), uncontrolled thyroid disease, or uncontrolled seizure disorders;
13. Hyponatremia (sodium <135 mmol/L) or a history of recurrent hyponatremia;
14. History of peptic ulcer disease or bleeding disorders;
15. Impaired liver function (ALT or AST > 100 IU/L or international normalized ratio (INR) >1.5), known cirrhosis, or liver transplantation;
16. Severe renal impairment (creatinine clearance <30 mL/min), history of renal transplantation, or undergoing dialysis;
17. Allergy or intolerance to duloxetine, pregabalin, or anesthetic agents;
18. Long-term use of gabapentin or pregabalin (regular use for more than three months);
19. Previous use of duloxetine or alternative serotonin-norepinephrine reuptake inhibitors (SNRIs), selective serotonin reuptake inhibitors (SSRIs), monoamine oxidase inhibitors (MAOIs), and tricyclic antidepressants;
20. A score >7 on the Hamilton Depression Rating Scale (HAMD);
21. Inability to complete study questionnaires;
22. Refusal to undergo randomization.

### 2.5. Randomization and allocation

To ensure the scientific rigor of the study design and minimize bias, the randomization scheme will be designed and supervised by an independent statistical expert who is not involved in patient recruitment or clinical management. The random allocation sequence will be generated using validated statistical software (SAS 9.4). Eligible patients will undergo a preoperative assessment for CS using the central sensitization inventory (CSI) as the evaluative instrument [46–48]. Patients with a CSI score of less than 40 will be assigned to Group 6, where they will receive the same intervention as Group 1. Patients with a CSI score of 40 or above will be randomly allocated to Groups 1–5 in a 1:1:1:1:1 ratio, with block sizes of 5 or 10 randomly chosen to reduce the predictability of allocation.

### 2.6. Blinding

This investigation is designed as a prospective, randomized, triple-blind clinical trial. Throughout the trial, all individuals involved in data collection, including patients, surgeons, clinical researchers, and statisticians, will strictly adhere to a blinding protocol regarding group allocations. The blinding will only be revealed once the final data analysis is completed. To ensure the blinding is maintained for all participants, including medical professionals and researchers, the subjects will receive capsule formulations of duloxetine and pregabalin, or placebos of equal appearance, in a random manner determined by a computer. The capsules have been carefully created to be identical in color, size, taste, smell, labeling, and packaging. This ensures that subjects are unable to determine the medication they are ingesting.

A designated staff member at the medical institution’s pharmacy will be responsible for dispensing duloxetine, pregabalin, and placebos to participants. They will have no knowledge of the specific group assignments. The medications will be distributed to participants according to a computer-generated randomization schedule, following the study protocol strictly. It is crucial to adhere to this process to ensure that each participant receives the study medication or placebo at the prescribed dosage and timing. The capsule count and administration intervals will be kept consistent across all the groups. The staff member will also meticulously document each participant’s medication compliance, which is essential for analyzing and interpreting the data later on.

The task of collecting, managing, and analyzing the data will be entrusted to a separate, unbiased team. This team will not be informed of the group assignments, ensuring the objectivity and reliability of the study results. The process of revealing the randomization and assignment will only occur once the required number of participants have been enrolled and the data analysis has been thoroughly completed. To validate the effectiveness of the blinding process, we will use bang’s blinding index (BBI) to assess the degree of blinding maintained throughout the trial [49]. After the intervention, patients will be asked to complete a blinded assessor questionnaire. This questionnaire will include a single question asking them to guess which treatment they received (such as “Guess Duloxetine,” “Guess Pregabalin,” “Guess the co-medication,” “Guess Placebo,” or “Do not Know”). This measure will help us evaluate how successful the blinding process is.

Data regarding participant treatment guesses and the calculated BBI will be reported descriptively in the Results section to quantitatively assess the effectiveness of blinding. These findings will be used to aid in the interpretation of the study outcomes and will be discussed in the context of potential study limitations in the Discussion section.

### 2.7. Treatment

#### 2.7.1. Usual care.

In this trial, a standardized protocol of general anesthesia will be administered to all participants who have given consent. All TKAs will be performed by the same surgeon (JBM). A midline skin incision, a standard medial parapatellar approach and a cemented total knee system (Smith & Nephew, London, UK) will be used in all patients. Additionally, an inflationary tourniquet will be applied at a pressure of 300 mm Hg to control bleeding during surgery.

A standardized multimodal analgesic protocol will be followed for all participants. As a preemptive analgesic strategy, patients will take 200 mg of celecoxib orally two hours before surgery. During the postoperative period, a patient-controlled analgesia (PCA) pump will be used. The pump will contain a 100 mL solution with 2,000 mg of fentanyl to meet personalized pain management needs. Once patients regain their ability to eat and drink again, they will be given a prescription for 1 mg of hydrocodone orally every 12 hours for a week to maintain continuous pain relief. Additionally, a combination therapy consisting of 200 mg of celecoxib, 37.5 mg of tramadol, and 650 mg of acetaminophen will be taken orally every 12 hours for the first six weeks after surgery. This combination aims to enhance pain relief through a synergistic approach using multiple drugs.

Prophylaxis against venous thromboembolic complications during the first week after surgery will involve administering a 20 mg subcutaneous injection of enoxaparin to all patients. Starting on the day after surgery, patients will be encouraged to walk with the help of a walker and to do a set of gradual range-of-motion exercises while still in bed, all under the guidance of medical staff. These activities are intended to facilitate a rapid recovery and reduce the chances of postoperative complications.

#### 2.7.2. Interventions.

**Fig 3:**
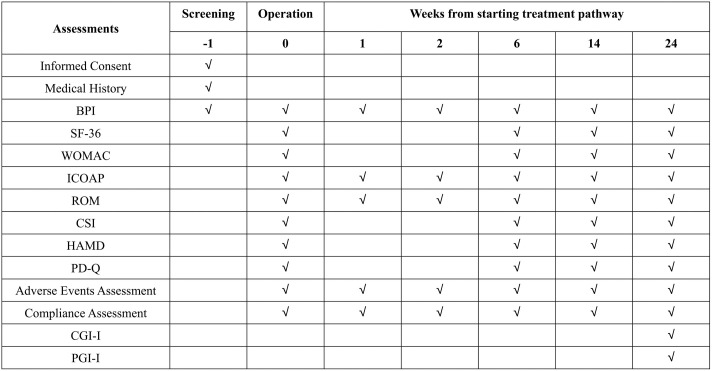
Study design. DLX: duloxetine; PGB: pregabalin.

This study will be structured into three distinct phases: an initial treatment phase that lasts six weeks, a high-dose/combination therapy phase that also lasts six weeks, and a two-week tapering-off period. The therapeutic protocol, described in Fig 3, will involve daily administration of 60 mg of duloxetine to patients in Groups 2 and 3, and 300 mg of pregabalin to those in Groups 4 and 5 during the initial treatment phase. Groups 1 and 6 will be given a placebo.

The transition to the high-dose/combination therapy phase will depend on evaluating the effectiveness of the treatment, which will be determined by changes in the brief pain inventory (BPI) 24-hour average pain score during the initial treatment period. Patients who experience a reduction in pain of over 30% will be considered “treatment responders” and their treatment will be stopped. Conversely, “non-responders,” who experience less than 30% pain relief, will continue to receive intensified therapy [43]. Group 2 will receive a daily dose of 120 mg of duloxetine, while Groups 3 and 4 will receive a combination of 60 mg of duloxetine and 300 mg of pregabalin daily. Group 5 will be treated with a daily dose of 600 mg of pregabalin. Non-responders in Groups 1 and 6 will remain on placebo. The dosages of the study drugs will be systematically reduced during the subsequent tapering period. Dosages will also be tapered for patients who discontinue treatment earlier.

In cases of significant intolerance to the prescribed dose levels, patients will be advised to decrease their dosage under the guidance of the investigating team for one week. The purpose will be to then gradually increase the dosage back to the originally prescribed level. If patients still experience intolerance after dosage adjustment, the study medication will be stopped to prioritize patient safety.

### 2.8. Withdrawal criteria

At any point during the study, participants will retain the freedom to withdraw their participation without needing to give a reason. This will not affect their relationship with healthcare providers. Participants can be assured that their decision to withdraw will not impact their access to medical care, their rights, or any follow-up procedures. There will be no negative consequences or penalties for withdrawing. In certain circumstances, such as concerns for patient safety or other significant factors, the chief investigator or regulatory authorities may decide to end a participant’s involvement in the clinical trial. To ensure the well-being of participants, individuals who are considering stopping their medication or withdrawing from the study will be strongly encouraged to consult with the research team for guidance on how to manage the discontinuation process.

### 2.9. Outcome measurement

Outcome metrics and patient characteristics will be systematically collected from electronic hospital information systems and medical charts. In addition, physical examinations will be conducted, and patients will be asked to complete questionnaires themselves.

#### 2.9.1. Primary outcomes.

The main goal of this study is to assess the severity of persistent pain in patients with CS six months after TKA. The aim is to determine whether high-dose monotherapy with either duloxetine or pregabalin, or their combined use, alongside a multimodal analgesic regimen, is effective in reducing pain. The choice of the six-month postoperative period as the assessment milestone is based on its significance in clinical practice for evaluating the overall success of TKA.

The BPI is a self-reported assessment tool used to measure both the severity of pain and its impact on functional ability [50]. Our research team has translated and cross-culturally adapted the BPI into Chinese. We have also validated the reliability and repeatability of the Chinese version of the BPI scale in Chinese patients with TKA [51,52]. The primary outcome measure is the 24-hour average pain score taken from the BPI. Patients will be asked to rate their average pain intensity over the past 24 hours using an 11-point scale ranging from 0 (no pain) to 10 (worst pain imaginable).

#### 2.9.2. Secondary outcomes.

In this study, we have meticulously designed the secondary outcome measures to comprehensively assess pain control and functional recovery in patients with CS following TKA. We will use various metrics to evaluate pain-related outcomes. These metrics include the cumulative consumption of PCA within 48 hours after the operation, as well as the timing and quantity of rescue analgesic consumption. To assess the severity of pain, we will use a battery of instruments, including the Western Ontario and McMaster Universities Osteoarthritis Index (WOMAC), the pain dimensions of the 36-Item Short Form Health Survey (SF-36), and the Intermittent and Constant Osteoarthritis Pain Questionnaire (ICOAP) [53–55].

Functional outcomes will be assessed by measuring the range of motion (ROM) of the knee joint, which includes both active (aROM) and passive (pROM) ROM. In addition, the WOMAC and SF-36 scores will be used to evaluate functional status. These assessments will be conducted at several predetermined follow-up time points using the appropriate evaluation tools.

To obtain a comprehensive understanding of the patients’ overall health condition and the effectiveness of the treatment, the study will gather data on various relevant factors. These factors include self-reported joint problems, CSI scores, HMAD scores, PainDETECT questionnaire (PD-Q) scores, Clinical Global Impression of Improvement (CGI-I) scores and Patient Global Impression of Improvement (PGI-I) scores [17,56,57]. The specific measurements that will be taken at each follow-up visit to evaluate the treatment’s efficacy and safety are illustrated in Fig 1.

### 2.10. Patient characteristics

In this investigation, the researchers aim to gather baseline descriptive data to assess the participants. The data collected will include age, gender, height (cm), weight (kg), body mass index (BMI), and education level. Additionally, this study will document the duration of OA pain symptoms and evaluate the severity of OA using the Kellgren-Lawrence (K-L) grading system. The consumption of medications related to OA pain will also be recorded. Furthermore, the health status of the participants will be assessed using the ASA Physical Status Classification System. Lifestyle factors, including smoking behaviors and alcohol intake patterns, will be duly considered. The researchers will document the participants’ medical history, existing comorbidities, medication history, and previous health issues to gain an understanding of their health status. All the data will be collected and processed confidentially to ensure participant privacy.

### 2.11. Safety

The safety evaluation will entail rigorous monitoring, recording, and reporting of all adverse events (AEs). The assessment will employ a combination of systematic and non-systematic methods. During each scheduled study visit in the six months following the procedure, participants will be systematically queried about preset common AEs related to the study medication using a standardized checklist. These AEs include drowsiness, insomnia, fatigue, dizziness, headache, dry mouth, decreased appetite, constipation, nausea, vomiting, peripheral edema, and diplopia [44]. Furthermore, all AEs reported spontaneously by participants or observed by investigators at any time, whether expected or related to the study treatment, will be thoroughly documented.

All recorded AEs will be coded using Medical Dictionary for Regulatory Activities (MedDRA, version 27) terminology, including Preferred Terms and System Organ Class. Serious adverse events (SAEs), defined as events leading to death, life-threatening experiences, prolonged hospitalization, or persistent/significant disability/incapacity, will be promptly reported to the principal investigator, ethics committee, and relevant regulatory authorities. The HAMD will be used at specific time points to monitor mental health status, including depressive symptoms and potential suicidal tendencies, as specific safety outcomes. Any clinically significant safety findings or events that may require discontinuation of treatment will be timely evaluated by the clinical team and managed according to best clinical practices and the study protocol.

In the final trial publication, the safety data report will include: (1) all SAEs, (2) AEs leading to treatment discontinuation, and (3) treatment-emergent adverse events (TEAEs) occurring in at least 5% of patients in any treatment group, presented by MedDRA System Organ Class and Preferred Term. A summary table detailing the overall incidence of AEs across treatment groups will also be provided. If appropriate, statistical tests may be performed to report differences in the frequency of key AEs between groups.

An interim analysis will be conducted after 50% of participants have completed the 6-month follow-up, using a group sequential design with an O’Brien-Fleming alpha-spending function to control the type I error rate at 0.05 [58]. The Data and Safety Monitoring Board (DSMB) will review interim results to assess both efficacy and futility. Based on emerging data, the DSMB may recommend trial continuation, modification, or early termination, even in the absence of formal stopping rules. Throughout the trial, the DSMB will monitor adverse events and efficacy data to ensure participant safety and ethical conduct. After the 12-month follow-up, participants in the monotherapy group will be offered access to the combination intervention if it is shown to be superior and no new contraindications arise.

### 2.12. Data collection and management

Trained personnel will collect demographic and baseline characteristic data during patient recruitment and registration (Table 1). Independent researchers will document clinical outcomes, survey responses, complication rates, and adverse events during hospitalization and follow-up. The researchers will follow strict data collection protocols to ensure accurate and reliable information. Additionally, an independent data monitoring committee will oversee the data collection process to maintain the quality and completeness of the data. No new participants will be enrolled during the re-evaluation phase of the study to avoid data confusion and potential bias. Each participant will receive a unique identification code to ensure patient privacy and data anonymity. Clinical and survey data will be collected and stored using this code. A patient identification checklist will link the data to the respective patients. The principal investigator will securely hold the key to access these codes, ensuring the security and confidentiality of the data.

**Table 1:** Baseline characteristics.

	Initial therapy						High-dose/Combination therapy					
	Group1 (n=)	Group2 (n=)	Group3 (n=)	Group4 (n=)	Group5 (n=)	Group6 (n=)	Group1 (n=)	Group2 (n=)	Group3 (n=)	Group4 (n=)	Group5 (n=)	Group6 (n=)
Age (years), mean (SD)												
Gender (F/M)												
BMI (kg/m^2^), mean (SD)												
Education level, n (%)												
Primary school												
High school												
Technical degree/ associate’s degree												
Bachelor’s degree												
Advanced/professional degree (MA, PhD, etc.)												
ASA grade (Ⅰ/Ⅱ/Ⅲ, n)												
K-L grade (Ⅲ/Ⅳ, n)												
Duration of OA pain symptoms(years), mean (SD)												
OA pain-related medication consumption, n (%)												
Acetaminophen												
NSAIDs												
Opioids												
Smoking consumption, n (%)												
Alcohol consumption, n (%)												
History of drug use, n (%)												
Comorbidities, n (%)												
Hypertension												
Diabetes												
Cardiac disease												
Cerebrovascular event												
Kidney disease												
Pulmonary disease												
Liver disease												

BMI: Body mass index; ASA: American society of anesthesiologists; K-L: Kellgren-Lawrence; OA: Osteoarthritis.

### 2.13. Sample size

The objective of this study is to evaluate the therapeutic effects of different treatment strategies on postoperative pain in patients with CS following TKA. Assuming a standard deviation (SD) value of 2.5, a two-sided test with 90% statistical power was used as the evaluation criterion. The study aims to detect a minimum difference of 1 point in the 24-hour average pain score on the BPI between patients subjected to high-dose monotherapy groups (Combination Groups 2 and 5) and those receiving combination therapy (Combination Groups 3 and 4) [43]. This 1-point threshold was chosen based on the methodological precedent established in the COMBO-DN trial, which employed the same effect size and analytic framework [43]. Furthermore, previous studies have shown that a 1-point change on the BPI scale (ranging from 0 to 10) represents the MCID for chronic pain conditions [59]. From the patient perspective, a 1-point reduction is considered a small but noticeable and meaningful improvement in pain intensity [60–62].

Sample size calculations are being carried out using the statistical software nQuery Advisor 7.0 (Statistical Solutions, Saugus, MA, USA). To ensure the validity of the analysis, each treatment group requires a minimum of 135 participants, as determined by these calculations. The clinical trial design takes into account the expected response rate to the treatment and the possibility of treatment discontinuation. It is anticipated that approximately 60% of participants will respond positively to the initial treatment, while an estimated 15% might discontinue treatment for various reasons during the study [43]. Based on these clinical scenarios, we intend to randomly assign a total of 1200 patients. This strategy aims to ensure that each treatment group has an adequate sample size for the data analysis phase, thereby enhancing the reliability and validity of the study’s findings.

Furthermore, multiple strategies will be implemented to maximize participant retention and minimize loss to follow-up, including: (1) a comprehensive informed consent process that clearly outlines study expectations and commitments; (2) regular follow-up contacts via participants’ preferred methods (clinic visits, phone calls, emails, or text messages); (3) flexible scheduling options for follow-up assessments; (4) appointment reminders; and (5) providing participants with contact information for a dedicated study coordinator to address any questions or concerns promptly. We will closely monitor the loss to follow-up rate throughout the entire trial process.

### 2.14. Statistical analysis

For the data analysis in this study, we will use IBM SPSS software (version 26.0, IBM Corp., New York, NY, USA). Continuous and descriptive numerical data will be reported as the means ± SDs, while categorical variables will be presented as frequencies and percentages. Statistical significance will be determined by a P value of less than 0.05. All analyses will adhere to the principles of intention-to-treat (ITT). The efficacy population for the analysis will include all patients who received treatment and had at least one post-baseline efficacy assessment during the high-dose monotherapy/combination therapy period. The safety population will consist of all patients who are randomly assigned and received treatment. The baseline assessment prior to the initiation of treatment will be used as the reference point, and the follow-up at six weeks post-surgery will mark the beginning of the high-dose monotherapy/combination therapy period.

The primary efficacy analysis will use a Mixed-Effects Model for Repeated Measures (MMRM) to compare changes in the 24-hour average pain score on the BPI between high-dose monotherapy and combination therapy. The MMRM will include fixed effects for treatment group (high-dose monotherapy vs. combination therapy), visit, treatment-by-visit interaction, initial treatment (duloxetine vs. pregabalin), baseline pain score, and baseline-by-visit interaction, with an unstructured covariance matrix. The MMRM does not include subject-level random effects. Within-subject correlations across visits are modeled using an unstructured covariance matrix to accommodate complex correlation patterns in longitudinal pain score data. Model fit will be assessed using likelihood ratio tests or information criteria, with alternative covariance structures will be considered if convergence issues arise. Results will report estimated means and 95% confidence intervals (CIs) for each treatment group, as well as the between-group mean difference, 95% CI, and corresponding *P*-value.

Secondary efficacy analyses will be conducted as exploratory measures. The efficacy of different treatments within each treatment period will be compared using the MMRM model. Safety outcomes will be summarized descriptively. The frequencies of patients experiencing TEAEs across different treatment approaches will be compared using Fisher’s exact test.

The MMRM, based on Maximum Likelihood Estimation (MLE), effectively utilizes all available observations under the Missing at Random (MAR) assumption [63,64]. To support this assumption, exploratory analyses will compare baseline characteristics such as age, sex, and baseline pain score between patients with and without missing data to evaluate missingness patterns. If the proportion of missing data for the primary efficacy endpoint (BPI 24-hour average pain score) exceeds 5%, multiple imputation (MI) will be conducted as a sensitivity analysis to assess the robustness of the findings [65]. The MI model will include all fixed-effect variables from the primary MMRM analysis and will generate 50 imputed datasets, selected based on Monte Carlo error convergence [66]. Each dataset will be analyzed using MMRM, and results will be pooled using Rubin’s rules to evaluate the impact of missing data on the primary conclusions [65,67]. To account for potential Missing Not at Random (MNAR) mechanisms, additional sensitivity analyses will be performed, including: (1) pattern-mixture models (PMM) stratified by treatment group or visit timepoint; (2) worst-case scenario analysis, where missing BPI scores are imputed as maximum pain scores to reflect treatment failure; and (3) adjusted MI models incorporating dropout reasons, such as adverse events or loss to follow-up, as covariates. These sensitivity analyses will be compared with the primary MMRM results to evaluate the robustness of conclusions under alternative missing data assumptions [64].

### 2.15. Cost-effectiveness analysis

To support clinical relevance and guide decision-making, a cost-effectiveness analysis (CEA) will be conducted from the perspective of the Chinese healthcare system, comparing high-dose duloxetine monotherapy, high-dose pregabalin monotherapy, and combination therapy for the treatment of postoperative chronic pain associated with central sensitization. The analysis will evaluate direct medical costs, such as medication use, adverse event management, follow-up consultations, and rehabilitation services, alongside treatment effectiveness measured in quality-adjusted life years (QALYs), derived from EQ-5D-5L utility scores [68]. A trial-based evaluation will be conducted using data collected over a 24-month period, during which costs and QALYs will be estimated for each intervention, stratified by central sensitization levels based on the CSI. Pain outcomes will be assessed using the 24-hour average pain score from the BPI, and generalized linear models will adjust for baseline covariates such as age, sex, and baseline pain score. Incremental cost-effectiveness ratios (ICERs) will be calculated to express the cost per QALY gained. To project long-term cost-effectiveness over a 20-year horizon, a Markov model will simulate patient transitions between defined health states, including pain relief, persistent pain, functional disability, re-treatment, and death, using annual cycles. Transition probabilities will be derived from trial data and supplemented by published literature on chronic pain progression. All future costs and QALYs will be discounted at an annual rate of 3%, and model calibration will be based on Chinese epidemiological and cost data [69]. To assess uncertainty, probabilistic sensitivity analysis (PSA) with 1,000 Monte Carlo simulations will be performed, with results presented as cost-effectiveness acceptability curves (CEACs). Additionally, one-way sensitivity analyses will be conducted to identify key cost-effectiveness drivers, and findings will be illustrated using Tornado diagrams. This comprehensive economic evaluation will provide essential evidence on the value for money of each treatment strategy, thereby informing resource allocation and clinical adoption in the context of China’s healthcare system.

### 2.16. Consent for publication

Written informed consent for publication of their clinical details and/or clinical images will be obtained from the participants. A copy of the consent form is available for review at any stage if required. Participation in this study is strictly confidential. Any information that is published will not reveal the identity of the participants.

## 3. Discussion

In recent years, duloxetine and pregabalin have been gradually accepted as analgesic agents for post-surgical pain management and have received positive feedback from both patients and clinicians. In gynecological and urological surgeries, duloxetine, which serves as a crucial component of multimodal analgesia protocols, has been proven to effectively enhance analgesic efficacy during the perioperative period while significantly reducing the consumption of opioid medications [70,71]. Similarly, preoperative oral administration of pregabalin has demonstrated remarkable outcomes in patients undergoing laparoscopic cholecystectomy, significantly alleviating postoperative pain and reducing the use of opioid analgesics [72]. These findings underscore the significance of duloxetine and pregabalin for postoperative analgesia.

Duloxetine effectively relieves musculoskeletal pain mediated by the CNS, including chronic pain associated with knee OA [30]. In patients with CS undergoing TKA, perioperative administration of duloxetine has been shown to reduce pain, decrease opioid usage, and enhance recovery without increasing the risk of adverse effects [31,32,73–75]. In the context of TKA, administering pregabalin during the perioperative period has been associated with a decreased risk of developing chronic neuropathic pain and a reduction in opioid consumption. This intervention has also been shown to improve joint mobility without increasing the incidence of adverse effects, such as nausea or vomiting [36,76,77]. Earlier studies have shown that the combination of duloxetine and pregabalin is an effective and safe therapy for reducing pain in patients with diabetic peripheral neuropathy and fibromyalgia [41–43]. This two-drug treatment produced positive therapeutic results without causing significant adverse effects, thus confirming its potential as a viable treatment option.

Residual pain is a significant contributor to patient dissatisfaction following TKA, and numerous studies have firmly established a robust correlation between preoperative CS and the occurrence of chronic postoperative pain, ultimately leading to decreased patient satisfaction. Duloxetine and pregabalin have gained some attention as off-label agents in the management of chronic pain. However, medical evidence on the optimal treatment for CS patients who undergo TKA and who experience suboptimal pain relief remains scarce-whether to increase the dose of a single agent or to transition to combination therapy. To fill this knowledge gap, we carefully designed the current clinical trial. Building on previous literatures, our study aims to be an advancement and improvement over similar RCTs conducted in the past. Our goal is to provide a more scientific and rigorous basis for the selection of postoperative pain treatment options for TKA and to develop a more precise and reliable approach to postoperative pain management for CS patients.

First, we will group and intervene in patients with CS while also establishing an additional blank control group (Group 6) comprising normal patients without CS. Both Group 6 and Group 1 will follow the same postoperative analgesia protocol to assess the impact of different CS statuses on the effectiveness of the routine postoperative pain relief regimen. Second, to evaluate pain improvement in patients with CS who experience inadequate relief after a 6-week treatment with 60 mg duloxetine or 300 mg pregabalin and subsequently receive an additional 6 weeks of treatment with 120 mg duloxetine or 600 mg pregabalin, the pain in Groups 2 and 5 will be compared at 6 months postoperatively. By conducting this comparison, we will be able to assess the effectiveness and safety of high-dose duloxetine and pregabalin in managing postoperative pain in this specific patient population. Third, we can evaluate the appropriateness of continuing high-dose monotherapy versus opting for a combination of both medications after suboptimal initial treatment responses by comparing pain outcomes between the high-dose monotherapy groups and the combination therapy groups at the six-month postoperative mark. This comparison enables a thorough evaluation of the effectiveness and safety of high-dose monotherapy and combination therapy approaches. Furthermore, this trial will present us with an opportunity to compare the effectiveness of standard dosages of duloxetine and pregabalin in managing postoperative pain among patients with CS undergoing TKA during the initial six-week treatment phase. Ultimately, during the postoperative follow-up period, we will use the CSI repeatedly to assess the level of CS in patients, with the aim of tracking its trends under different interventions.

This study has several limitations that warrant consideration. First, it does not control for certain variables known to influence the experience and reporting of chronic pain, such as gender differences and psychosocial factors. Although randomization is expected to balance these confounders across groups to some extent, future larger-scale studies with stratified or covariate-adjusted designs are needed to further delineate their impact. Second, the study is conducted at a single high-volume tertiary orthopedic center in Northwest China, which serves a diverse patient population from multiple provinces. However, site-specific influences such as institutional protocols, urban healthcare infrastructure, and regional demographic characteristics may still limit the generalizability of the findings to other healthcare settings. As such, the generalizability of the findings to other healthcare settings, particularly rural or community-based hospitals, may be limited. Multicenter trials across varied clinical contexts are necessary to validate and extend the applicability of these results. Third, all surgeries in this trial are performed by a single experienced surgeon. While this approach enhances internal consistency and minimizes variability in surgical technique, it may limit the external validity of the findings when applied to broader clinical settings involving surgeons with varying expertise levels. Fourth, the study adopts relatively strict inclusion and exclusion criteria to enhance internal validity and participant safety, especially given the elderly population involved and the high-dose pharmacologic interventions being tested. However, this restrictiveness may reduce the applicability of the results to real-world populations, where comorbidities and medication use patterns are more heterogeneous. Future pragmatic trials with broader eligibility criteria or real-world evidence studies are warranted to support translation into clinical practice. Fifth, the primary outcome relies on self-reported pain assessment using the BPI. While the BPI is a validated and widely accepted tool in both clinical and research settings, self-reported measures may be subject to recall bias, especially in older adults who may experience subtle cognitive changes. Although patients with overt cognitive impairment were excluded at baseline, minor deficits may still influence pain reporting and should be considered when interpreting the results. Lastly, Furthermore, since all participants in this study will be elderly, their tolerance to high doses of duloxetine or pregabalin may be compromised, potentially leading to an underestimation of the dropout rate. However, previous research has shown that high-dose duloxetine is effective and well-tolerated in treating pain associated with diabetic peripheral neuropathy [29], and combined pregabalin and duloxetine therapy has improved clinical outcomes in patients with fibromyalgia [42]. Additionally, combined pregabalin and duloxetine therapy has been found to be well-tolerated for diabetic neuropathic pain and can improve pain relief in patients with inadequate pain control under monotherapy [41], and both high-dose duloxetine and pregabalin monotherapy or in combination are well-tolerated in chronic pain conditions [43]. Therefore, based on existing research, our study team believes that the participants could tolerate the required drug doses without significantly impacting the dropout rate. Despite these limitations, this trial will provide important evidence regarding optimal pharmacologic strategies for managing chronic postsurgical pain in older adults following TKA, particularly in patients with features of central sensitization. The findings will help inform both individualized treatment approaches and future large-scale confirmatory studies.

To ensure that this study is scientific and rigorous, we will implement a comprehensive approach to randomization, blinding, and allocation concealment, fulfilling the criteria for a true triple-blind, randomized controlled trial. Additionally, we will strictly adhere to the Consolidated Standards of Reporting Trials (CONSORT) guidelines for conducting and reporting the study [78]. This trial aims to provide scientifically sound and rigorous guidance for postoperative analgesic medication in patients with CS undergoing TKA.

**Trial status:** The protocol version number is V1.1 and the date of this version is 13 March, 2024. The anticipated starting date of recruitment is 1 August 2024. Recruitment is expected to be completed in December 2026.

## Supporting information

S1 File Research plan. (DOC)

S2 File SPIRIT Fillable checklist 15-Aug-2013. (DOC)

## References

[1] Hawker GA, Bohm E, Dunbar MJ, Jones CA, Noseworthy T, Marshall DA, et al. The Effect of Patient Age and Surgical Appropriateness and Their Influence on Surgeon Recommendations for Primary TKA: A Cross-Sectional Study of 2,037 Patients. J Bone Joint Surg Am. 2022;104(8):700–8. doi: 10.2106/JBJS.21.00597 35226616

[2] Lee SH, Kim DH, Lee YS. Is there an optimal age for total knee arthroplasty?: A systematic review. Knee Surg Relat Res. 2020;32(1):60. doi: 10.1186/s43019-020-00080-1 33198817 PMC7667791

[3] Harris WH, Sledge CB. Total hip and total knee replacement (1). N Engl J Med. 1990;323(11):725–31. doi: 10.1056/NEJM199009133231106 2201916

[4] Zhu S, Qian W, Jiang C, Ye C, Chen X. Enhanced recovery after surgery for hip and knee arthroplasty: a systematic review and meta-analysis. Postgrad Med J. 2017;93(1106):736–42. doi: 10.1136/postgradmedj-2017-134991 28751437 PMC5740550

[5] Oussedik S, Abdel MP, Victor J, Pagnano MW, Haddad FS. Alignment in total knee arthroplasty. Bone Joint J. 2020;102-B(3):276–9. doi: 10.1302/0301-620X.102B3.BJJ-2019-1729 32114811

[6] Cross WW 3rd, Saleh KJ, Wilt TJ, Kane RL. Agreement about indications for total knee arthroplasty. Clin Orthop Relat Res. 2006;446:34–9. doi: 10.1097/01.blo.0000214436.49527.5e 16672869

[7] Lee YS, Howell SM, Won Y-Y, Lee O-S, Lee SH, Vahedi H, et al. Kinematic alignment is a possible alternative to mechanical alignment in total knee arthroplasty. Knee Surg Sports Traumatol Arthrosc. 2017;25(11):3467–79. doi: 10.1007/s00167-017-4558-y 28439636

[8] Auyong DB, Allen CJ, Pahang JA. Reduced length of hospitalization in primary total knee arthroplasty P. n.d. 10.1016/j.arth.2015.05.007 26024988

[9] Jauregui JJ, Cherian JJ, Pierce TP. Long-term survivorship and clinical outcomes following total knee arth. 2023. 10.1016/j.arth.2015.05.052 26100473

[10] Scott CEH, Howie CR, MacDonald D, Biant LC. Predicting dissatisfaction following total knee replacement: a prospec. n.d. 10.1302/0301-620X.92B9.24394 20798443

[11] Bourne RB, Chesworth BM, Davis AM, Mahomed NN, Charron KDJ. Patient satisfaction after total knee arthroplasty: who is satisfied and who is not?. Clin Orthop Relat Res. 2010;468(1):57–63. doi: 10.1007/s11999-009-1119-9 19844772 PMC2795819

[12] Gunaratne R, Pratt DN, Banda J, Fick DP, Khan RJK, Robertson BW. Patient Dissatisfaction Following Total Knee Arthroplasty: A Systematic Review of the Literature. J Arthroplasty. 2017;32(12):3854–60. doi: 10.1016/j.arth.2017.07.021 28844632

[13] Woolf CJ. Central sensitization: implications for the diagnosis and treatment of pain. Pain. 2011;152(3 Suppl):S2–15. doi: 10.1016/j.pain.2010.09.030 20961685 PMC3268359

[14] Woolf CJ, Chong MS. Preemptive analgesia--treating postoperative pain by preventing the establishment of central sensitization. Anesth Analg. 1993;77(2):362–79. doi: 10.1213/00000539-199377020-00026 8346839

[15] Yunus MB. Central sensitivity syndromes: a new paradigm and group nosology for fibromyalgia and overlapping conditions, and the related issue of disease versus illness. Semin Arthritis Rheum. 2008;37(6):339–52. doi: 10.1016/j.semarthrit.2007.09.003 18191990

[16] Hochman JR, Gagliese L, Davis AM, Hawker GA. Neuropathic pain symptoms in a community knee OA cohort. Osteoarthritis Cartilage. 2011;19(6):647–54. doi: 10.1016/j.joca.2011.03.007 21440077

[17] Hochman JR, Davis AM, Elkayam J, Gagliese L, Hawker GA. Neuropathic pain symptoms on the modified painDETECT correlate with signs of central sensitization in knee osteoarthritis. Osteoarthritis Cartilage. 2013;21(9):1236–42. doi: 10.1016/j.joca.2013.06.023 23973136

[18] Valdes AM, Suokas AK, Doherty SA, Jenkins W, Doherty M. History of knee surgery is associated with higher prevalence of neuropathic pain-like symptoms in patients with severe osteoarthritis of the knee. Semin Arthritis Rheum. 2014;43(5):588–92. doi: 10.1016/j.semarthrit.2013.10.001 24188720

[19] Murphy SL, Lyden AK, Phillips K, Clauw DJ, Williams DA. Association between pain, radiographic severity, and centrally-mediated symptoms in women with knee osteoarthritis. Arthritis Care Res (Hoboken). 2011;63(11):1543–9. doi: 10.1002/acr.20583 22034116 PMC3205461

[20] Wluka AE, Yan MK, Lim KY, Hussain SM, Cicuttini FM. Does preoperative neuropathic-like pain and central sensitisation affect the post-operative outcome of knee joint replacement for osteoarthritis? A systematic review and meta analysis. Osteoarthritis Cartilage. 2020;28(11):1403–11. doi: 10.1016/j.joca.2020.07.010 32791103

[21] Lundblad H, Kreicbergs A, Jansson KA. Prediction of persistent pain after total knee replacement for osteoarthritis. J Bone Joint Surg Br. 2008;90(2):166–71. doi: 10.1302/0301-620X.90B2.19640 18256082

[22] Kim MS, Koh IJ, Sohn S, Kang BM, Kwak DH, In Y. Central Sensitization Is a Risk Factor for Persistent Postoperative Pain and Dissatisfaction in Patients Undergoing Revision Total Knee Arthroplasty. J Arthroplasty. 2019;34(8):1740–8. doi: 10.1016/j.arth.2019.03.042 30992238

[23] Wylde V, Palmer S, Learmonth ID, Dieppe P. The association between pre-operative pain sensitisation and chronic pain after knee replacement: an exploratory study. Osteoarthritis Cartilage. 2013;21(9):1253–6. doi: 10.1016/j.joca.2013.05.008 23973138

[24] Nijs J, George SZ, Clauw DJ, Fernández-de-Las-Peñas C, Kosek E, Ickmans K, et al. Central sensitisation in chronic pain conditions: latest discoveries and their potential for precision medicine. Lancet Rheumatol. 2021;3(5):e383–92. doi: 10.1016/S2665-9913(21)00032-1 38279393

[25] Knadler MP, Lobo E, Chappell J, Bergstrom R. Duloxetine: clinical pharmacokinetics and drug interactions. Clin Pharmacokinet. 2011;50(5):281–94. doi: 10.2165/11539240-000000000-00000 21366359

[26] Fields HL, Heinricher MM, Mason P. Neurotransmitters in nociceptive modulatory circuits. Annu Rev Neurosci. 1991;14:219–45. doi: 10.1146/annurev.ne.14.030191.001251 1674413

[27] Basbaum AI, Fields HL. Endogenous pain control systems: brainstem spinal pathways and endorphin circuitry. Annu Rev Neurosci. 1984;7:309–38. doi: 10.1146/annurev.ne.07.030184.001521 6143527

[28] Woolf CJ, American College of Physicians, American Physiological Society. Pain: moving from symptom control toward mechanism-specific pharmacologic management. Ann Intern Med. 2004;140(6):441–51. doi: 10.7326/0003-4819-140-8-200404200-00010 15023710

[29] Lunn MPT, Hughes RAC, Wiffen PJ. Duloxetine for treating painful neuropathy, chronic pain or fibromyalgia. Cochrane Database Syst Rev. 2014;2014(1):CD007115. doi: 10.1002/14651858.CD007115.pub3 24385423 PMC10711341

[30] Chappell AS, Ossanna MJ, Liu-Seifert H, Iyengar S, Skljarevski V, Li LC, et al. Duloxetine, a centrally acting analgesic, in the treatment of patients with osteoarthritis knee pain: a 13-week, randomized, placebo-controlled trial. Pain. 2009;146(3):253–60. doi: 10.1016/j.pain.2009.06.024 19625125

[31] Ho K-Y, Tay W, Yeo M-C, Liu H, Yeo S-J, Chia S-L, et al. Duloxetine reduces morphine requirements after knee replacement surgery. Br J Anaesth. 2010;105(3):371–6. doi: 10.1093/bja/aeq158 20573635

[32] Koh IJ, Kim MS, Sohn S, Song KY, Choi NY, In Y. Duloxetine Reduces Pain and Improves Quality of Recovery Following Total Knee Arthroplasty in Centrally Sensitized Patients: A Prospective, Randomized Controlled Study. J Bone Joint Surg Am. 2019;101(1):64–73. doi: 10.2106/JBJS.18.00347 30601417

[33] Field MJ, Cox PJ, Stott E, Melrose H, Offord J, Su T-Z, et al. Identification of the alpha2-delta-1 subunit of voltage-dependent calcium channels as a molecular target for pain mediating the analgesic actions of pregabalin. Proc Natl Acad Sci U S A. 2006;103(46):17537–42. doi: 10.1073/pnas.0409066103 17088553 PMC1859964

[34] Memtsoudis SG, Poeran J, Zubizarreta N, Cozowicz C, Mörwald EE, Mariano ER, et al. Association of Multimodal Pain Management Strategies with Perioperative Outcomes and Resource Utilization: A Population-based Study. Anesthesiology. 2018;128(5):891–902. doi: 10.1097/ALN.0000000000002132 29498951

[35] Quintero L, Cardenas R, Suarez-Roca H. Stress-induced hyperalgesia is associated with a reduced and delayed GABA inhibitory control that enhances post-synaptic NMDA receptor activation in the spinal cord. Pain. 2011;152(8):1909–22. doi: 10.1016/j.pain.2011.04.017 21636214

[36] Buvanendran A, Kroin JS, Della Valle CJ, Kari M, Moric M, Tuman KJ. Perioperative oral pregabalin reduces chronic pain after total knee arthroplasty: a prospective, randomized, controlled trial. Anesth Analg. 2010;110(1):199–207. doi: 10.1213/ANE.0b013e3181c4273a 19910619

[37] Derry S, Bell RF, Straube S, Wiffen PJ, Aldington D, Moore RA. Pregabalin for neuropathic pain in adults. Cochrane Database Syst Rev. 2019;1(1):CD007076. doi: 10.1002/14651858.CD007076.pub3 30673120 PMC6353204

[38] Moore RA, Straube S, Wiffen PJ, Derry S, McQuay HJ. Pregabalin for acute and chronic pain in adults. Cochrane Database Syst Rev. 2009;(3):CD007076. doi: 10.1002/14651858.CD007076.pub2 19588419 PMC4167351

[39] Pritchett YL, McCarberg BH, Watkin JG, Robinson MJ. Duloxetine for the management of diabetic peripheral neuropathic pain: response profile. Pain Med. 2007;8(5):397–409. doi: 10.1111/j.1526-4637.2007.00305.x 17661853

[40] Sultan A, Gaskell H, Derry S, Moore RA. Duloxetine for painful diabetic neuropathy and fibromyalgia pain: systematic review of randomised trials. BMC Neurol. 2008;8:29. doi: 10.1186/1471-2377-8-29 18673529 PMC2529342

[41] Tesfaye S, Sloan G, Petrie J, White D, Bradburn M, Julious S, et al. Comparison of amitriptyline supplemented with pregabalin, pregabalin supplemented with amitriptyline, and duloxetine supplemented with pregabalin for the treatment of diabetic peripheral neuropathic pain (OPTION-DM): a multicentre, double-blind, randomised crossover trial. Lancet. 2022;400(10353):680–90. doi: 10.1016/S0140-6736(22)01472-6 36007534 PMC9418415

[42] Gilron I, Chaparro LE, Tu D, Holden RR, Milev R, Towheed T, et al. Combination of pregabalin with duloxetine for fibromyalgia: a randomized controlled trial. Pain. 2016;157(7):1532–40. doi: 10.1097/j.pain.0000000000000558 26982602

[43] Tesfaye S, Wilhelm S, Lledo A, Schacht A, Tölle T, Bouhassira D, et al. Duloxetine and pregabalin: high-dose monotherapy or their combination? The “COMBO-DN study”--a multinational, randomized, double-blind, parallel-group study in patients with diabetic peripheral neuropathic pain. Pain. 2013;154(12):2616–25. doi: 10.1016/j.pain.2013.05.043 23732189

[44] Gilron I, Baron R, Jensen T. Neuropathic pain: principles of diagnosis and treatment. Mayo Clin Proc. 2015;90(4):532–45. doi: 10.1016/j.mayocp.2015.01.018 25841257

[45] Chan A-W, Tetzlaff JM, Gøtzsche PC, Altman DG, Mann H, Berlin JA, et al. SPIRIT 2013 explanation and elaboration: guidance for protocols of clinical trials. BMJ. 2013;346:e7586. doi: 10.1136/bmj.e7586 23303884 PMC3541470

[46] Neblett R, Cohen H, Choi Y, Hartzell MM, Williams M, Mayer TG, et al. The Central Sensitization Inventory (CSI): establishing clinically significant values for identifying central sensitivity syndromes in an outpatient chronic pain sample. J Pain. 2013;14(5):438–45. doi: 10.1016/j.jpain.2012.11.012 23490634 PMC3644381

[47] Tanaka K, Nishigami T, Mibu A, Manfuku M, Yono S, Shinohara Y, et al. Validation of the Japanese version of the Central Sensitization Inventory in patients with musculoskeletal disorders. PLoS One. 2017;12(12):e0188719. doi: 10.1371/journal.pone.0188719 29216211 PMC5720706

[48] Mayer TG, Neblett R, Cohen H, Howard KJ, Choi YH, Williams MJ, et al. The development and psychometric validation of the central sensitization inventory. Pain Pract. 2012;12(4):276–85. doi: 10.1111/j.1533-2500.2011.00493.x 21951710 PMC3248986

[49] Bang H, Ni L, Davis CE. Assessment of blinding in clinical trials. Control Clin Trials. 2004;25(2):143–56. doi: 10.1016/j.cct.2003.10.016 15020033

[50] Cleeland CS, Ryan KM. Pain assessment: global use of the Brief Pain Inventory. Ann Acad Med Singap. 1994;23(2):129–38. 8080219

[51] Wang S, Yao S, Wei J, Shang L, Xu C, Ma J. Psychometric Properties of the Brief Pain Inventory Among Patients With Osteoarthritis Undergoing Total Knee Arthroplasty Surgery. J Arthroplasty. 2024;39(3):672–6. doi: 10.1016/j.arth.2023.08.072 37648099

[52] Wang S, Yao S, Shang L, Xu C, Ma J. Validation of the Chinese version of the Brief Pain Inventory in patients with knee osteoarthritis. J Orthop Surg Res. 2023;18(1):720. doi: 10.1186/s13018-023-04218-1 37742029 PMC10518095

[53] Davis AM, Lohmander LS, Wong R, Venkataramanan V, Hawker GA. Evaluating the responsiveness of the ICOAP following hip or knee replacement. Osteoarthritis Cartilage. 2010;18(8):1043–5. doi: 10.1016/j.joca.2010.04.013 20435154

[54] Laucis NC, Hays RD, Bhattacharyya T. Scoring the SF-36 in Orthopaedics: A Brief Guide. J Bone Joint Surg Am. 2015;97(19):1628–34. doi: 10.2106/JBJS.O.00030 26446970 PMC5029523

[55] Giesinger JM, Hamilton DF, Jost B, Behrend H, Giesinger K. WOMAC, EQ-5D and Knee Society Score Thresholds for Treatment Success After Total Knee Arthroplasty. J Arthroplasty. 2015;30(12):2154–8. doi: 10.1016/j.arth.2015.06.012 26160647

[56] Zimmerman M, Martinez JH, Young D, Chelminski I, Dalrymple K. Severity classification on the Hamilton Depression Rating Scale. J Affect Disord. 2013;150(2):384–8. doi: 10.1016/j.jad.2013.04.028 23759278

[57] Kahlenberg CA, Nwachukwu BU, McLawhorn AS, Cross MB, Cornell CN, Padgett DE. Patient Satisfaction After Total Knee Replacement: A Systematic Review. HSS J. 2018;14(2):192–201. doi: 10.1007/s11420-018-9614-8 29983663 PMC6031540

[58] Ciolino JD, Kaizer AM, Bonner LB. Guidance on interim analysis methods in clinical trials. J Clin Transl Sci. 2023;7(1):e124. doi: 10.1017/cts.2023.552 37313374 PMC10260346

[59] Reed DE 2nd, Stump TE, Monahan PO, Kroenke K. Comparable Minimally Important Differences and Responsiveness of Brief Pain Inventory and PEG Pain Scales across 6 Trials. J Pain. 2024;25(1):142–52. doi: 10.1016/j.jpain.2023.07.028 37544394 PMC10859144

[60] Salaffi F, Stancati A, Silvestri CA, Ciapetti A, Grassi W. Minimal clinically important changes in chronic musculoskeletal pain intensity measured on a numerical rating scale. Eur J Pain. 2004;8(4):283–91. doi: 10.1016/j.ejpain.2003.09.004 15207508

[61] Yarlas A, Miller K, Wen W, Lynch SY, Munera C, Pergolizzi JV Jr, et al. Buprenorphine transdermal system compared with placebo reduces interference in functioning for chronic low back pain. Postgrad Med. 2015;127(1):38–45. doi: 10.1080/00325481.2014.992715 25526229

[62] Angst F, Aeschlimann A, Michel BA, Stucki G. Minimal clinically important rehabilitation effects in patients with osteoarthritis of the lower extremities. J Rheumatol. 2002;29(1):131–8. 11824949

[63] Graham JW. Missing data analysis: making it work in the real world. Annu Rev Psychol. 2009;60:549–76. doi: 10.1146/annurev.psych.58.110405.085530 18652544

[64] White IR, Royston P, Wood AM. Multiple imputation using chained equations: Issues and guidance for practice. Stat Med. 2011;30(4):377–99. doi: 10.1002/sim.4067 21225900

[65] Jakobsen JC, Gluud C, Wetterslev J, Winkel P. When and how should multiple imputation be used for handling missing data in randomised clinical trials - a practical guide with flowcharts. BMC Med Res Methodol. 2017;17(1):162. doi: 10.1186/s12874-017-0442-1 29207961 PMC5717805

[66] Graham JW, Olchowski AE, Gilreath TD. How many imputations are really needed? Some practical clarifications of multiple imputation theory. Prev Sci. 2007;8(3):206–13. doi: 10.1007/s11121-007-0070-9 17549635

[67] Tan P-T, Cro S, Van Vogt E, Szigeti M, Cornelius VR. A review of the use of controlled multiple imputation in randomised controlled trials with missing outcome data. BMC Med Res Methodol. 2021;21(1):72. doi: 10.1186/s12874-021-01261-6 33858355 PMC8048273

[68] Zhou T, Guan H, Wang L, Zhang Y, Rui M, Ma A. Health-Related Quality of Life in Patients With Different Diseases Measured With the EQ-5D-5L: A Systematic Review. Front Public Health. 2021;9:675523. doi: 10.3389/fpubh.2021.675523 34268287 PMC8275935

[69] Attema AE, Brouwer WBF, Claxton K. Discounting in Economic Evaluations. Pharmacoeconomics. 2018;36(7):745–58. doi: 10.1007/s40273-018-0672-z 29779120 PMC5999124

[70] Castro-Alves LJ, Oliveira de Medeiros ACP, Neves SP, Carneiro de Albuquerque CL, Modolo NS, De Azevedo VL, et al. Perioperative Duloxetine to Improve Postoperative Recovery After Abdominal Hysterectomy: A Prospective, Randomized, Double-Blinded, Placebo-Controlled Study. Anesth Analg. 2016;122(1):98–104. doi: 10.1213/ANE.0000000000000971 26421810

[71] Kotecha P, Sahai A, Malde S. Use of Duloxetine for Postprostatectomy Stress Urinary Incontinence: A Systematic Review. Eur Urol Focus. 2021;7(3):618–28. doi: 10.1016/j.euf.2020.06.007 32605820

[72] Sarakatsianou C, Theodorou E, Georgopoulou S, Stamatiou G, Tzovaras G. Effect of pre-emptive pregabalin on pain intensity and postoperative morphine consumption after laparoscopic cholecystectomy. Surg Endosc. 2013;27(7):2504–11. doi: 10.1007/s00464-012-2769-3 23344509

[73] Yang J-M, Wang Y, Li J-Y, Li C-C, Wang Z-T, Shen Z, et al. Duloxetine for rehabilitation after total knee arthroplasty: a systematic review and meta-analysis. Int J Surg. 2023;109(4):913–24. doi: 10.1097/JS9.0000000000000230 37097617 PMC10389646

[74] Kim MS, Koh IJ, Sung YG, Park DC, Na JW, In Y. Preemptive Duloxetine Relieves Postoperative Pain and Lowers Wound Temperature in Centrally Sensitized Patients Undergoing Total Knee Arthroplasty: A Randomized, Double-Blind, Placebo-Controlled Trial. J Clin Med. 2021;10(13):2809. doi: 10.3390/jcm10132809 34202314 PMC8269433

[75] YaDeau JT, Mayman DJ, Jules-Elysee KM, Lin Y, Padgett DE, DeMeo DA, et al. Effect of Duloxetine on Opioid Use and Pain After Total Knee Arthroplasty: A Triple-Blinded Randomized Controlled Trial. J Arthroplasty. 2022;37(6S):S147–54. doi: 10.1016/j.arth.2022.02.022 35346549

[76] Dong J, Li W, Wang Y. The effect of pregabalin on acute postoperative pain in patients undergoing total knee arthroplasty: A meta-analysis. Int J Surg. 2016;34:148–60. doi: 10.1016/j.ijsu.2016.08.521 27573693

[77] Sawan H, Chen AF, Viscusi ER, Parvizi J, Hozack WJ. Pregabalin reduces opioid consumption and improves outcome in chronic pain patients undergoing total knee arthroplasty. Phys Sportsmed. 2014;42(2):10–8. doi: 10.3810/psm.2014.05.2053 24875968

[78] Moher D, Hopewell S, Schulz KF, Montori V, Gøtzsche PC, Devereaux PJ, et al. CONSORT 2010 explanation and elaboration: updated guidelines for reporting parallel group randomised trials. BMJ. 2010;340:c869. doi: 10.1136/bmj.c869 20332511 PMC2844943

